# FFPred 3: feature-based function prediction for all Gene Ontology domains

**DOI:** 10.1038/srep31865

**Published:** 2016-08-26

**Authors:** Domenico Cozzetto, Federico Minneci, Hannah Currant, David T. Jones

**Affiliations:** 1 Bioinformatics Group, Department of Computer Science, University College London, Gower Street, London, WC1E 6BT, UK

## Abstract

Predicting protein function has been a major goal of bioinformatics for several decades, and it has gained fresh momentum thanks to recent community-wide blind tests aimed at benchmarking available tools on a genomic scale. Sequence-based predictors, especially those performing homology-based transfers, remain the most popular but increasing understanding of their limitations has stimulated the development of complementary approaches, which mostly exploit machine learning. Here we present FFPred 3, which is intended for assigning Gene Ontology terms to human protein chains, when homology with characterized proteins can provide little aid. Predictions are made by scanning the input sequences against an array of Support Vector Machines (SVMs), each examining the relationship between protein function and biophysical attributes describing secondary structure, transmembrane helices, intrinsically disordered regions, signal peptides and other motifs. This update features a larger SVM library that extends its coverage to the cellular component sub-ontology for the first time, prompted by the establishment of a dedicated evaluation category within the Critical Assessment of Functional Annotation. The effectiveness of this approach is demonstrated through benchmarking experiments, and its usefulness is illustrated by analysing the potential functional consequences of alternative splicing in human and their relationship to patterns of biological features.

Thanks to a combination of experimental assays and computational studies, knowledge about protein function has been steadily accumulating in public databases, where it is commonly described through the Gene Ontology^1^ (GO). On the one hand, hypothesis-driven research has traditionally led to the thorough characterization of one or few proteins at a time. On the other hand, high-throughput technologies have opened the way to very large-scale exploratory surveys to study biological processes, identify binding partners, or establish subcellular locations. Meanwhile, some homology-based approaches for annotation transfers have developed enough to produce fairly confident results. The GO consortium, for instance, makes wide use of a semi-automated tool for phylogenetic analysis and functional inference^2^, and of mappings between protein domain families to GO terms that are valid for all their members^3^. Despite these multi-pronged efforts, however, a substantial fraction of deposited sequences still have no functional annotation at all, and the remaining ones usually lack assignments for at least one GO domain. When available, this information may not be at the finest level of detail possible, not only because of the way some electronically inferred annotations are generated, but also because of the varying levels of resolution characterizing experimental results^4^,^5^. Finally, nature can still spring surprises: protein moonlighting demonstrates that novel functions can still await discovery even for well-researched proteins^6^.

One way to fill in some of these gaps employs machine learning to examine diverse biological data types separately or in combination, and to provide functional hypotheses that complement homology-based annotation transfers^7^,^8^,^9^. In particular, over the years several supervised methods have been devised for function prediction from amino acid sequences, which are easier to collect than structural data or genome-wide measurements of gene expression or protein-protein interactions. GOStruct^10^ and FANN-GO^11^, for instance, make GO term assignments by analysing the patterns of BLAST^12^ E-values to experimentally characterized proteins using structured Support Vector Machines (SVM) and multioutput neural networks, respectively. Given the computational complexity of training classifiers with multiple correlated outputs, it is difficult to learn the relationship between the input features and the whole GO; the proponents have therefore adopted workarounds such as reducing the number of output terms and ensemble modelling. Rather than tackling this complex structured learning problem, other researchers have tested with success the possibility of converting it into a set of simpler binary classification tasks. This approach has recently allowed our group to train GO term-specific neural networks from features describing the results of profile-profile comparisons^13^.

Alignment-derived features, such as similarity scores, sequence coverage and E-values, can help learn which sequence similarity patterns correlate with the conservation of individual annotations, thus allowing more effective control on homology-based annotation transfers. Complementary efforts have investigated the usefulness of biophysical attributes to make homology-free inferences, under the assumption that proteins with similar functions would have similar biological features despite the lack of significant sequence similarities. For example, the occurrence of signal peptides gives useful hints about protein subcellular location, and also limits the number of their molecular functions and of the biological processes they partake. The idea was first implemented in ProtFun, which is based on neural networks trained for the functional classification of protein sequences from similarities in amino acid composition, and content of signal peptides, trans-membrane helices, post-translationally modified residues as well as other biological features^14^,^15^. The observation that the length and position of intrinsically disordered protein regions strongly correlates with some molecular activities and biological processes led to an expanded set of sequence-derived features, which FFPred scans through a library of GO term-specific SVMs to annotate protein chains^16^,^17^. A more recent study has confirmed the effectiveness of this feature-based approach with the use of random forests for supervised learning^18^.

In this paper, we describe the latest FFPred release, which updates the previous one with an extended vocabulary spanning all three GO domains, reflecting the increasing attention in cellular component annotations, as evidenced from recent experiments in the Critical Assessment of Functional Annotation initiative. We evaluate FFPred 3 prediction accuracy using two complementary approaches and describe its improvements over the previous version. Finally, we show how its predictions can help get a glimpse into the effects of alternative splicing on human protein function. The results show patterns of functional conservation and variation consistent with the presence or absence of particular biophysical attributes and with general biological knowledge.

## Results and Discussion

### Summary of tool updates

**Figure 1:**
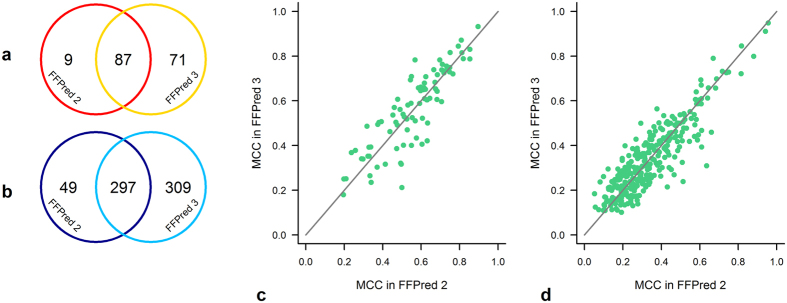
Comparison between FFPred 2 and FFPred 3. Extent of the overlap between FFPred 2 and FFPred 3 GO term lists in the MF (**a**) and BP (**b**) domains. Most common terms in the MF (**c**) and BP (**d**) sub-ontologies are expected to be predicted with similar accuracy, as measured by the MCC.

Thanks to the continued growth of annotation databases, the latest FFPred release features a GO term vocabulary, which spans all three GO domains for the first time and is almost twice the size of that in the previous update. Supplementary Data file 1 lists the 868 GO terms, for which a dedicated SVM is available along with the classification accuracy estimated from the validation experiments following the training procedures. The new release makes still use of SVMs, which are known to successfully handle imbalanced classification tasks–typical in computational biology–where it is extremely important to allow for error control and avoid overfitting to known observations. Subcellular localization prediction has been the focus of many previous studies, which mostly focused on the well-known compartments of eukaryotic cells–such as nucleus, cytosol, endoplasmic reticulum, Golgi apparatus, mitochondrion and other organelles. The newly added cellular component terms in FFPred 3 also include some of the numerous macromolecular complexes found in them. The extensions to the other two sub-ontologies provide more specific descriptions for functional categories previously covered, and they reflect the increasing body of knowledge in areas such as organelle localization, immune system and reproductive processes, response to stimuli and chromosome segregation. A small fraction of molecular function and biological process terms have been removed (Fig. 1a,b), because they no longer occur in curated databases–mostly after the GO consortium made them obsolete. The majority of functional categories that have been retained can be predicted with negligible changes in expected accuracy–though some exceptions exist. As a consequence of the extended knowledge about human protein function since the last update, the patterns of biophysical attrbutes linked to terms such as sulfur compound metabolic process (GO:0006790), neurotrophin TRK receptor signaling pathway (GO:0048011), growth factor activity (GO:0008083) and protein kinase binding (GO:0019901) can be more easily identified and modelled. For other functions, such as calcium ion transport (GO:0006816), single organismal cell-cell adhesion (GO:0016337), ATPase activity (GO:0016887), and nuclease activity (GO:0004518), SVM performance has dropped, suggesting that their relationships to sequence-derived features are more complex than previously appreciated (Fig. 1c,d).

The tool is designed with a focus on the function of human proteins, and so annotations curated for other organisms are never used for training. To learn effectively the relationship between biophysical attributes and GO terms, sufficiently large numbers of positive instances are needed, thus limiting the specificity of the functional categories that can be currently predicted. While this feature may not be desirable for all applications, its benefits to overcome some well-known limitations of homology-based annotation transfers have already been reported^15^,^17^. Interestingly, previous work showed that the tool can also help annotate protein function for other eukaryotic organisms. The updated tool is publicly available on the web at http://bioinf.cs.ucl.ac.uk/ffpred.

### Performance evaluation

The accuracy estimates in Supplementary Data file 1 are GO term-specific and point out the usefulness of FFPred 3 to prioritize human genes for downstream experimental screening when homology offers little or no help. To complement this analysis and gauge how well protein function as a whole can be predicted for such difficult cases, a timed experiment similar to the Critical Assessment of Functional Annotation challenge was conducted, by training a separate SVM library using the public databases released in November 2013. The resulting 597 classifiers were then used to assign GO terms to human proteins with no experimentally verified biological roles at that time, and their accuracy was finally measured against the UniProtKB-GOA data as of March 2016. For comparison purposes under difficult working conditions with limited or completely missing homology information, additional predictions were generated by a baseline method (Naïve), which ranks GO terms by prevalence in UniProtKB-GOA, and by a sequence similarity-based approach (BLAST), which can transfer annotations only from distantly related and experimentally characterized proteins as detailed in Methods. Other machine-learning based tools for GO term prediction from patterns of biological features could not be included in the study: ProtFun^15^ has not been updated in a very long time and only covers a handful of currently valid GO terms, whereas ProFET^18^ requires training from scratch classifiers for all GO categories of interest.

**Figure 2:**
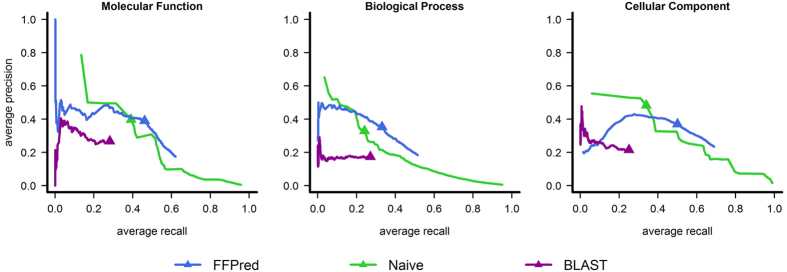
Graphical summary of the precision–recall analysis. The three panels show the evaluation results for the MF (left), BP (centre) and CC (right) domains, respectively. The full triangles mark the points associated with the maximum F-measure.

The precision-recall plots in Fig. 2 and the data in Table 1 provide graphical and numerical reports on the evaluation results for the three separate GO domains, according to standard practice in the field. At high levels of recall (i.e. above roughly 40% for molecular function and 20% for the other two sub-ontologies), FFPred 3 predictions achieve higher precision values than the baseline approaches do, and the maximum F-scores in Table 1 clearly back up this observation. However, the highest scoring predictions made by BLAST for subcellular locations and by Naïve for all sub-ontologies attain higher precision than the corresponding ones by FFPred 3. This result surprisingly suggests that these less sophisticated approaches are more useful than FFPred 3, when only a handful of assays can be run on each protein. Or are they?

It is widely accepted that an obvious pitfall of precision-recall analysis is the total disregard of how informative predictions are. The most confident GO term assignments made by Naïve for each test protein–GO:0043226 (binding), GO:0005488 (organelle) and GO:0009987 (cellular process)–are far from useful in cutting down the options for the design of experiments, indeed. Nonetheless, their very shallow nature guarantees that they will be eventually confirmed for most, if not all, proteins. Furthermore, comparing the precision values achieved by different methods and plotted against the same level of recall could be more ambiguous than it looks at first sight. If the recall is less than 1.0, the predictors are evaluated on non-identical sets of target proteins, which can even be disjoint. Another confounding aspect is the number of GO term predictions above a given decision threshold made for individual proteins: predictors based on high-throughput functional data aim at high recall and generally produce longer lists of assignments than those generated by methods based on homology transfers, which tend to achieve higher precision. Finally, correctly assigning the term *t* to distinct proteins *p* and *q* can pose prediction challenges of diverse nature, depending on how many proteins are annotated with *t*, and on how closely *p* and *q* follow the patterns of features used to build the classifiers–e.g. sequence similarity, domain architecture, biological attributes, gene expression and so on. Therefore, it is useful to look at method performance from a different angle, by considering both the accuracy and the informativeness of equal numbers of high scoring predictions for each target and sub-ontology–thus reducing the above biases and yielding results that can be interpreted more clearly and more easily by non-specialists, too.

**Figure 3:**
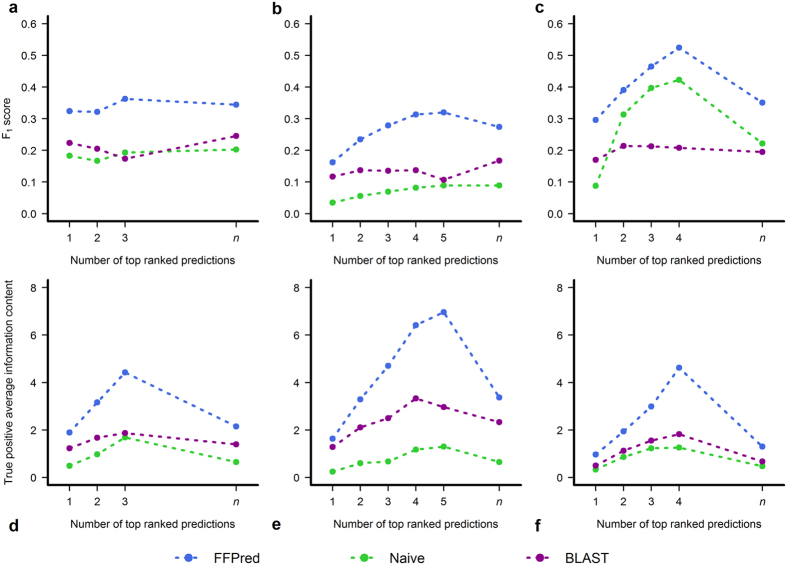
Comparison of the prediction accuracy and informativeness against number of top ranked predictions. The graphs on the top row compare the average F-measure of the highest scoring GO term assignments made by FFPred 3, Naïve and BLAST for the MF (left), BP (centre) and CC (right) domains, respectively. The bottom row shows the average information content of the true positives for the same predictions in the top row. Data are plotted only when there are at least 25 targets with *x* ∈ {1, 2, 3, 4, 5} predictions and 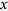 validated annotations or more. The label *n* represents the case where for each protein the number of predictions assessed equals the number of experimentally supported functions.

The top row panels in Fig. 3 summarize prediction quality in terms of F_1_ measure and the underlying precision and recall values are plotted in Figure S1. It is quite clear that FFPred 3 is superior to both Naïve and BLAST across all three GO domains, because it achieves higher recall than the other predictors do, in combination with intermediate values of precision. The data also clearly confirm the expectation that Naïve predictions generally are highly precise, but not deep enough in the GO graph to outperform the other approaches in terms of recall. The results for the CC sub-ontology are an interesting exception: the low numbers of false negatives most likely arise from the relatively shorter distances between nodes associated with experimental annotations and nodes associated with the most frequent terms in UniProtKB-GOA. The plots also clearly illustrate the limits of homology-based transfers in such challenging situations. When the evolutionary distances from previously annotated proteins are large, only the most general functional aspects are retained (e.g. catalytic or transporter activity), while the finer details diverge (e.g. the nature of the substrates and the chemistry of the reactions), thus resulting in high numbers of both false positives and false negatives, and ultimately affecting negatively precision, recall and F-measure values.

As mentioned above, the design and implementation of FFPred 3 produced a list of GO terms with varying levels of detail, so it could be questioned how informative its predictions are and how helpful they can be to experimenters. In Fig. 3, the plots in the bottom row show the average amount of useful information the highest scoring predictions would actually provide. For this purpose, the analysis only considers true positive predictions, which are not regarded as equally valuable as in the standard precision-recall analysis, however. They are rather weighted according to their information content, which estimates their specificity and informativeness from their occurrence in the UniProtKB/SwissProt database – so that more frequent functional categories are down-weighted, and vice versa. The plots undoubtedly prove that FFPred 3 correct predictions are consistently more specific than those generated by BLAST, which in turn are more specific than those made by Naïve. Therefore, despite the relatively low levels of term specificity, FFPred 3 can give useful hints to drive the experimental characterization of proteins, when routes alternative to homology transfers are needed. Table S1 gives some clear examples of how well FFPred 3 top-ranked predictions compare with the validated GO term assignments, which some proteins with no prior experimental functional data have recently acquired.

### Insights into the functional consequences of alternative splicing in humans

Experimentally supported functional information for individual splice variants is generally scarce–only a handful of isoform-level GO term annotations have been reviewed and included in public databases. Even when some isoforms encoded by the same gene have been assayed, the data are still largely incomplete, because the experiments are usually focussed on a particular functional aspect. Within this active area of research, FFPred 3 and similar methods for protein function prediction have the opportunity to help investigate the functional ramifications of alternative splicing. Indeed, very often comparative sequence analysis can only suggest that the relatively small sequence changes between splice isoforms cause more or less pronounced structural and functional differences. In other words, this approach is typically unable to put forward more detailed testable hypotheses. This opens up the possibility that alternative splicing products may not encode biochemically active molecules, but rather constitute a reservoir for natural selection^19^,^20^,^21^–a conjecture that is also hard to verify. Notwithstanding, experimental evidence shows that the functional divergence between alternative splice variants can vary from subtle modulations of biochemical activities to completely antagonistic regulatory roles^22^. It is therefore interesting to investigate: *i*) which functional aspects tend to be more robust to splicing, and consequently conserved across splice variants of the same gene; and *ii*) whether canonical isoforms tend to be enriched in functions that are different from those over-represented in their alternative variants–see Methods for further details on the conservation and *primarity* scores.

To examine these patterns, a large-scale survey was carried out on 9,214 human proteins and their recorded splice variants using FFPred 3, under the assumption that eventually they all fulfil a physiological role in the cell. The analysis was restricted to the GO term predictions compatible with the manually curated assignments existing in UniProtKB/SwissProt, as to reduce the effects of spurious results on the biological interpretation. The summary data in Supplementary Data file 2 indicate that the GO terms used in this study display varying levels of conservation across sets of alternatively spliced transcripts, even though it is difficult to assess the statistical significance of the observed differences. Only five predicted (and admittedly broad) functions appear to be consistently assigned to all the variants of a gene, and very few of them are highly conserved, when the focus is on the most reliably predicted GO terms–i.e. the SVM Matthews correlation coefficient value is in the top 50% of the distribution recorded for the corresponding sub-ontology. For instance, only six of such terms annotate all isoforms of a gene in 90% or more of the cases examined. Therefore, despite the use of a consolidated set of predictions, the findings support the expectation that alternative splicing plays a role in diversifying the cellular functional repertoire. Support for this theory is strengthened by the differential associations of individual biological roles with canonical or alternative splice isoforms – as gauged by the GO term *primarity* scores. The Supplementary Data file 3 indicate that there are many more GO categories preferentially associated with principal variants than with alternative ones, partly because these analyses are restricted to predicted functions in line with available annotations in UniProtKB/SwissProt. Nevertheless, the GO terms with high *primarity* scores tend to represent more constitutive cellular functions, and those with negative scores appear to be mostly associated with larger sets of alternatively spliced genes or to be induced by changes in the environment or in the cellular conditions. As mentioned above, it is difficult to draw statistically sound conclusions from this initial study: identifying the canonical isoform of each gene is still an open question, and here a rather simple and pragmatic approach was taken just like in previous studies.

**Figure 4:**
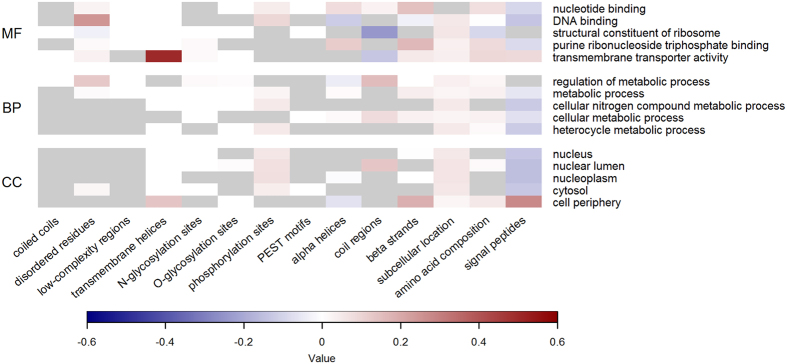
Enrichment of biological features for the most conserved functions in human alternative isoforms. The heatmaps compare the feature values calculated for the annotated splice variants with those used to train the classifiers for each of the five most conserved and confidently predicted functions in the MF (top), BP (centre) and CC (bottom) domain, respectively. The classifiers are in the top 50% of the corresponding sub-ontology. Warmer (colder) colours represent median feature group values that are higher (lower) in the human isoform proteome than in the positive training set for the corresponding GO term. Grey cells indicate feature groups not used by FFPred 3 to make predictions.

**Figure 5:**
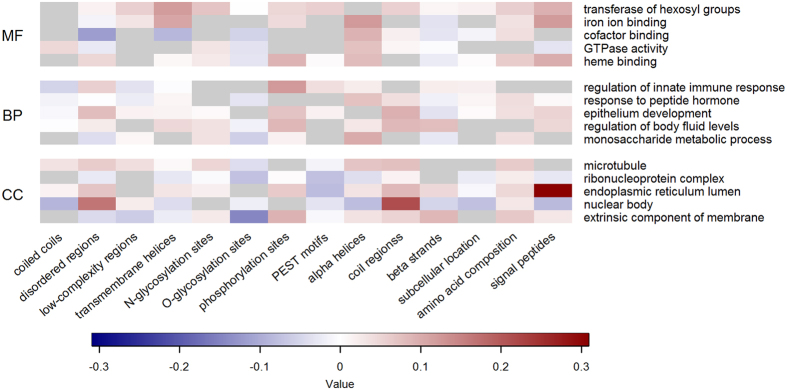
Relationship between biological features and GO terms preferentially associated with main splice isoforms. For each function, the heatmaps report the correlation between the feature values calculated for the annotated splice variants with the estimated probability produced by the corresponding classifier. Only the five GO terms with highest expected accuracy and propensity for the canonical splice variants are listed for the MF (top), BP (centre) and CC (bottom) domain, respectively. The classifiers are in the top 50% of the corresponding sub-ontology. Warmer (colder) colours represent higher (lower) values of median correlation across each feature group. Grey cells indicate feature groups not used by FFPred 3 to make predictions.

**Figure 6:**
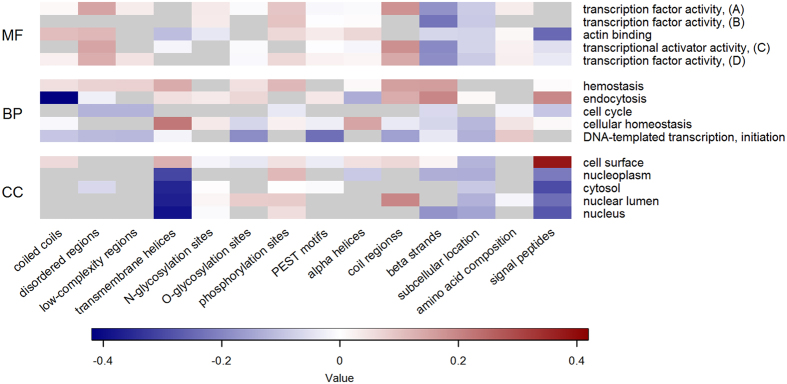
Relationship between biological features and GO terms preferentially associated with alternative splice isoforms. For each function, the heatmaps report the correlation between the feature values calculated for the annotated splice variants with the estimated probability produced by the corresponding classifier. Only the five GO terms with highest expected accuracy and propensity for alternative variants are listed for the MF (top), BP (centre) and CC (bottom) domain, respectively. The classifiers are in the top 50% of the corresponding sub-ontology. Warmer (colder) colours represent higher (lower) values of median correlation in each feature group. Grey cells indicate feature groups not used by FFPred 3 to make predictions. Some GO names have been abbreviated: RNA polymerase II core promoter proximal region sequence-specific DNA binding transcription factor activity involved in positive regulation of transcription (transcription factor activity (**A**); RNA polymerase II core promoter proximal region sequence-specific DNA binding transcription factor activity (transcription factor activity (**B**); RNA polymerase II transcription regulatory region sequence-specific DNA binding transcription factor activity involved in positive regulation of transcription (transcriptional activator activity (**C**); sequence-specific DNA binding RNA polymerase II transcription factor activity (transcription factor activity (**D**).

To emphasize the unique advantages that analyzing biological features can offer, Fig. 4 gives some insight into their relationship with some of the most conserved functions in each GO domain–see Methods for more details. The heatmap allows to link the over- and under-representation of specific biophysical attributes with the conservation of particular functional aspects. Similarly, Figs 5 and 6 show the extent of positive or negative correlation between sequence-derived feature groups and the GO terms that are preferentially associated with principal or alternative splice variants, respectively. The results generally reflect well-established trends between functional categories and the occurrence or lack of intrinsically disordered residues, transmembrane helices and signal peptides, and these interpretable patterns of association also apply to extended lists of GO terms, which are either expected to be predicted with lower confidence or to be less conserved (Figures S2, S3 and S4).

The figures above provide a general overview across the whole human isoform proteome; however, the online server allows to study how alternative splicing is likely to preserve or abolish individual functions, by providing a detailed graphical view of the biological features detected in the input sequences. The following showcases how functional conservation and variation are consistent with the presence or absence of particular biophysical attributes and, most importantly, with independent biological knowledge.

Protein intrinsic disorder has long been linked to binding activities and regulatory processes in the light of both experimental and computational investigations^23^,^24^,^25^, and its enrichment in DNA binding proteins has a two-fold explanation. Basic leucine zipper (bZIP) and AT hook domains–both well known examples of disordered regions–are frequently found in many transcription factors and regulators, and some are conserved in their splice isoforms, too. The proto-oncogene c-Fos (UniProt accession P01100) and the high mobility group protein HMGI-C (UniProt accession P52926) include one bZIP and three AT hook motifs, respectively, which are all conserved across their known splice isoforms. Most often, however, DNA binding proteins usually include additional disordered segments that are not directly involved in DNA binding, but rather in the establishment of transient and highly specific protein-protein interactions for transactivation purposes. These regions are either maintained upon splicing–like the C-terminal domain of c-Fos–or swapped with other disordered segments to rewire cellular and signaling networks^26^.

Signal peptides and transmembrane helices provide useful hints about protein subcellular localization and transmembrane transporter activities. They are unsurprisingly over and under-represented accordingly in those splice isoforms that need to retain the corresponding roles. The main and alternative isoforms of both the calcium-transporting ATPase type 2C member 1 (UniProt accession P98194) and of the 5-hydroxytryptamine receptor 3E (UniProt accession A5X5Y0) clearly illustrate this point. Alternative splicing hardly affects the transmembrane segments of these channels–only the isoform P98194-2 loses one helix–therefore they still localize in the membrane, and likely act as transporters of possibly different molecules.

Some associations–such as those between beta strands and several functional categories–may not look blatantly obvious, but brief scrutiny reveals their consistency with known biological facts. Nucleotides such as FAD, NAD and NADP are commonly bound by βαβ super-secondary structure motifs, which usually occur in tandem in the Rossman fold where they can form relatively large beta sheets. Mitochondrial glutathione reductase (UniProt accession P00390) has five known isoforms that all preserve the nucleotide binding site, for instance, thus suggesting that the sequence differences do not impact this functional aspect, but something else. It is known that the isoform P00390-1 is indeed found in the mithocondrion, while isoform P00390-2 is cytoplasmatic, for instance. The enrichment of residues in beta strands in isoforms at the cell periphery is also easily explained by the abundance of immunoglobulin-like (Ig-like) domains, which fold into a beta sandwich structure and are involved in a wide range of functions such as cell surface recognition, immune response and muscle structural organization. Both the mucosal addressin cell adhesion molecule 1(UniProt accession Q13477) and the leukocyte Ig-like receptor subfamily A member 5 (UniProt accession A6NI73) exemplify well this over-representation. Both proteins include a signal peptide followed by two Ig-like domains, one transmembrane helix and a C-terminal cytosolic region. All recorded splicing events cause the removal or replacement of sequence regions outside the signal peptide and the core of the Ig-like domains, thus proving that the alternative variants are still secreted.

Based on these examples, we would expect that this updated version of FFPred 3 will assist experimentalists narrow down the number of assays to functionally characterize individual variants of their own interest. In turn, those efforts will definitely stimulate further bio-curation work to interpret this information and make it available in machine-readable format. Initial computational studies have been carried out to advance this area of functional genomics using gene expression profile data^27^,^28^; their integration with other complementary sources of biological information that are tissue and condition-specific will undoubtedly be the focus of many more investigations in the near future.

## Methods

### Datasets and procedures for training and testing

Training procedures employed the term definitions and relationships defined in the GO^1^ OBO flat file released on 2015-02-03, the annotations for human proteins in UniProt-GOA^29^ released on 2015-04-02 and in UniProtKB^30^ release 2015_02, and the UniRef90^31^ release 2015_02 for sequence similarity searches. GO term-specific Support Vector Machines (SVM) were trained as detailed before^17^, and the following is a brief overview of the procedure, which is also graphically summarised in Supplementary Figure S5. Candidate functional classes were identified based on the availability of sufficiently large and confident positive and negative instances, which were split into training (70%) and validation (30%) data. The training subset was then encoded through 258 sequence-derived features covering a range of 14 different functional and structural aspects; the resulting vectors were fed into SVM-Light^32^ to perform feature selection and parameter optimization. Based on the number of training instances available for each function, the number of folds *k* ranges between 3 and 5, within the constraint that the partitions are equally sized. Feature selection was performed using a backward elimination approach, which involves first using all feature groups to estimate classification accuracy, and then iteratively testing if the removal of each feature group improves it. At each step, a grid search of the SVM hyper-parameter space was conducted with *k*-fold cross-validation to estimate SVM performance using the highest average Matthews correlation coefficient (MCC)


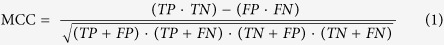


where TP is the number of proteins correctly labelled as positives (true positives); TN is the number of proteins correctly labelled as negatives (true negatives); FP is the number of misclassified negative cases (false positives); and FN is the number of misclassified positive instances (false negatives). These parameters were used to build a binary classifier from all training examples, the performance of which was tested against the proteins in the unseen validation set. Only GO terms corresponding to predictors achieving MCC ≥ 0.05 were retained, and for them FFPred 3 makes predictions with SVMs trained on the joint training and validation sets to make the most of available annotations. Platt scaling^33^ is applied to estimate the posterior probability that the input protein performs the function associated with a SVM given the raw output score.

### Datasets and procedures for performance evaluation

Only for the purpose of estimating prediction accuracy, an intermediate version of the SVM library was trained using the GO OBO flat file released on 2013-11-05, the UniProt-GOA gene association file for human submitted to the GO Consortium on 2013-10-28, UniProtKB and UniRef90 release 2013_10. The training procedures outlined above produced a vocabulary consisting of 400 terms in the biological process (BP) domain, 108 in the molecular function (MF) domain, and 89 in the cellular component (CC) domain, which allowed to make predictions for all human protein sequences released as targets of the second Critical Assessment of Functional Annotation challenge^34^. The benchmark set was collated from the UniProt-GOA gene association file, by selecting those human proteins that received GO term assignments supported by evidence code EXP, IDA, IMP, IGI, IEP, TAS or IC between 2014-01-20 (end of the CAFA2 prediction stage) and 2016-03-14 (the database release date). Annotations to the term “protein binding” (GO:0005515) were discarded because they convey limited functional information unless the context is quoted (e.g. where and when the activity takes place and the requirement or absence of other molecules), and because these qualifiers are neglected by current function prediction evaluation protocols. This resulted in 3,881 annotations for 1,365 proteins in total–602 MF annotations for 454 proteins, 1,802 BP annotations for 661 proteins, and 1,477 CC annotations for 991 proteins.

Prediction accuracy was measured separately for each GO domain by precision-recall analysis as in similar studies following the lead of the CAFA experiments^34^,^35^. For each protein *x* in the benchmark set and decision threshold *v*, the set of predicted terms *P*_*x,v*_ was built by collecting all terms with confidence scores greater than or equal to v and their ancestors in GO linked by “is a” relationships and different from the root; the set of reference terms *R*_*x*_ was generated in a similar way by up-propagating the validated annotations for *x*. These sets were used to calculate the number of true positives *tp*_*x,v*_, false positives *fp*_*x,v*_ and false negatives *fn*_*x,v*_ respectively as the sizes of the intersection *P*_*x,v*_ ∩ *R*_*x*_, of the set difference *P*_*x,v*_\*R*_*x*_ and of the set difference *R*_*x*_\*P*_*x,v*_. These data were combined into precision


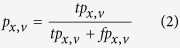


and recall


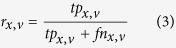


and then averaged across the test set using the formulas


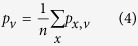



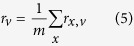


where *m* is the number of target proteins in the GO domain at hand and *n* is the number of those with at least one prediction scoring at least *v*. Finally, the average F-measure for the threshold *v* was calculated as


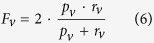


that is by taking the harmonic mean of 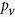 and 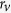.

A complementary evaluation of function prediction quality was carried out on the top-ranked predictions for each target *t* and GO domain *d*. To this end, after ranking based on confidence scores, the initial predictions were trimmed to the same length *l* ∈ {1, 2, 3, 4, 5, *n*_*t*,*d*_}, where *n*_*t*,*d*_ is the number of experimental annotations for *t* in the sub-ontology *d*. To handle ties in confidence scores, first 1,000 prediction lists of the desired length *l* were randomly sampled without replacement for each protein. Then, the average values of precision, recall, F-measure were calculated for each list of top *l* predictions; finally the average of such statistics over all replicates were analysed.

Along with the above statistics, the average sum of true positive information content was also calculated from all replicates. The information content of a GO term *t* was estimated in a Bayesian framework as proposed by Clark and Radivojac^36^ using the equation


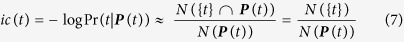


where ***P**(t)* represents the set of parent nodes of *t*, and the function *N*(·) returns for any set of GO terms the number of human proteins annotated in UniProtKB-SwissProt with evidence code EXP, IDA, IMP, IGI, IEP, TAS or IC.

The Supplementary Data file 4 includes the complete sets of reference annotations and of predictions used in these performance comparison experiments.

### Baseline function prediction methods

Naïve predictions were generated based on the frequency of the GO term annotations for human sequences recorded in UniProt-GOA as of 2013-10-28. To this end, initial counts were obtained for all GO terms except “protein binding” (GO:0005515) supported by the evidence codes EXP, IDA, IPI, IMP, IGI, IEP, IC and TAS. The data were then propagated following “is a” links in the GO released on 2013-11-05, and finally scaled between 0 and 1 for each domain separately, by dividing the final counts by the number of occurrences of the root node and rounding the result to three decimals like FFPred does. The resulting 6,504 pairs of GO terms (469 for CC, 1,268 for MF and 4,767 for BP) and scores were used to annotate all proteins in the benchmark set.

BLAST predictions were obtained by first collecting all BLAST^12^ hits in the UniRef90^31^ sequence database released in October 2013 with an E-value greater than 1e-03. Then the annotations in UniProtKB release 2013_10 supported by evidence code EXP, IDA, IPI, IMP, IGI, IEP, IC and TAS were transferred to the target sequences. GO term confidence scores were calculated by dividing the local alignment sequence identity by 100. When multiple BLAST hits were annotated with the same function, the highest score was retained.

### Annotation and functional analysis of human splice variants

The sequences of the human isoform proteome and the classification between main and alternative splice variants were obtained from the release 2015_03 of UniProtKB/SwissProt and the accompanying “varsplic” file. Individual isoforms were discarded if a) their amino acid sequence is unknown; or b) it is shorter than 15 amino acids; or c) it is longer than 1500 amino acids, or d) it includes non-standard amino acid symbols; or e) it is recorded in a separate database entry due to substantial differences from the canonical sequence. When these filters led to the exclusion of main variants, associated alternative sequences were removed from the dataset as well. This initial screening yielded 28,310 splice variants for 9,267 UniProtKB/SwissProt entries.

FFPred 3 was run to make isoform-specific GO term predictions, which were then screened for consistency with the UniProtKB/SwissProt data. Only functional classes that were either explicitly assigned by the curators or implied by the GO data released on 2015-02-27 were retained. Removal of principal isoforms at this stage also led to the elimination of all related alternative variants, hence producing a final dataset *P*_*as*_ consisting of 28,142 sequences for 9,214 UniProtKB/SwissProt entries.

Patterns of conservation and variation were analysed for all GO terms predicted to the splice isoforms of at least 20 distinct UniProtKB/SwissProt entries. For each functional class *G*, the survey aimed at quantifying its tendency to be conserved upon splicing, as well as its preference for principal rather than alternative splice variants. The average conservation of *G* across splice variants of the same gene was measured as the ratio between the number of UniProtKB/SwissProt entries where *G* was assigned to all isoforms, and the number of database records where it was predicted for at least one isoform. The *primarity* of *G–*that is its enrichment among main isoforms rather than alternative variants–was taken as


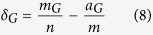


where *m*_*G*_ and *a*_*G*_ are respectively the numbers of main and alternative isoforms annotated with *G*, while *n* = 9214 is the number of genes in the dataset, and *m* = 28142 is the total number of splice variants. Therefore, *δ*_*G*_ > 0 if *G* is preferentially found among canonical isoform predictions; *δ*_*G*_ < 0 if *G* is assigned more often to alternative variants than to main ones; and *δ*_*G*_ = 0 if *G* is equally associated with the two sets of protein products.

To investigate further and interpret the conservation of each GO term *g* in the light of current biological knowledge, the biological attributes associated with the set of canonical and splicing variants annotated with *g (V*_*g*_) were compared with those previously observed in the positive training set (*T*_*g*_) of the corresponding SVM. In particular, for each sequence-derived feature *f*, the median value *m*_*g*,*f*,*T*_ observed during the training process was compared to *m*_*g*,*f*,*V*_–the median value in *V*_*g*_–by first mapping the latter to the lowest percentile 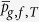 seen in *T*_*g*_ and then by calculating


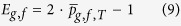


Therefore, *E*_*g*,*f*_ = 0 if the two median values are identical, *E*_*g*,*f*_ > 0 if on average *f* takes higher values in *V*_*g*_ than in *T*_*g*_, while *E*_*g*,*f*_ < 0 if *f* typically has lower values in *V*_*g*_ than *T*_*g*_. Similarly, the association between a feature *f* and a functional class *g* that is over-represented in either set of canonical or alternative protein isoforms was estimated using Pearson’s correlation coefficient between the values *f* takes on *V*_*g*_ and the correspondin_*g*_ SVM output scores.

## Additional Information

**How to cite this article**: Cozzetto, D. *et al.* FFPred 3: feature-based function prediction for all Gene Ontology domains. *Sci. Rep.* **6**, 31865; doi: 10.1038/srep31865 (2016).

## Supplementary Material

Supplementary Information

Supplementary Data 1

Supplementary Data 2

Supplementary Data 3

Supplementary Data 4

## Figures and Tables

**Table 1:** Performance comparison between FFPred 3 and the baseline prediction methods.

GO domain	Method	Threshold	TP	FP	FN	NP	Precision	Recall	F_1_
MF	FFPred	0.581	1443	3457	1818	427	0.390	0.461	0.422
	BLAST	0.210	952	5740	2309	216	0.266	0.282	0.274
	Naïve	0.152	1081	1643	2180	454	0.397	0.391	0.394
BP	FFPred	0.576	5792	13013	14469	655	0.353	0.331	0.342
	BLAST	0.203	5272	83543	14989	345	0.173	0.271	0.211
	Naïve	0.273	4136	8423	16125	661	0.329	0.241	0.278
CC	FFPred	0.730	3800	7424	4576	985	0.369	0.500	0.425
	BLAST	0.204	2030	15655	6346	422	0.215	0.251	0.232
	Naïve	0.579	2869	3077	5507	991	0.483	0.340	0.399

For each method, the table reports the total numbers of true positives (TP), false positives (FP) and false negatives (FN) each method achieves at the decision threshold that maximises the F_1_ score for each GO domain. NP is the number of proteins with at least one prediction with a confidence score greater than or equal to the corresponding threshold value, which is used to calculate the average precision of each method according to equation (4) in the main text. The average recall is calculated using equation (5) using the number of proteins with annotations in the GO domain under consideration, which can be found in the section “Methods”. The latter two values are used to locate the full triangles in the precision-recall space shown in Fig. 2.
