# Supplementary material for: FFPred 3: feature-based function prediction for all Gene Ontology domains
Source: Sci Rep. 2016 Aug 26;6:31865. doi: 10.1038/srep31865 (PMC4999993; doi:10.1038/srep31865)
Supplement: Supplementary Information [file srep31865-s1.doc]

Supplementary information

FFPred 3: feature-based function prediction for all Gene Ontology domains

Domenico Cozzetto^1*^, Federico Minneci^1*^, Hannah Currant^1^ and David T. Jones^1§^

^1^Bioinformatics Group, Department of Computer Science, University College London, Gower Street, London, WC1E 6BT, UK

^*^ Equal contribution to the study

^§^Corresponding author

E-mail: [d.t.jones@ ucl.ac.uk](mailto:d.t.jones@ ucl.ac.uk)


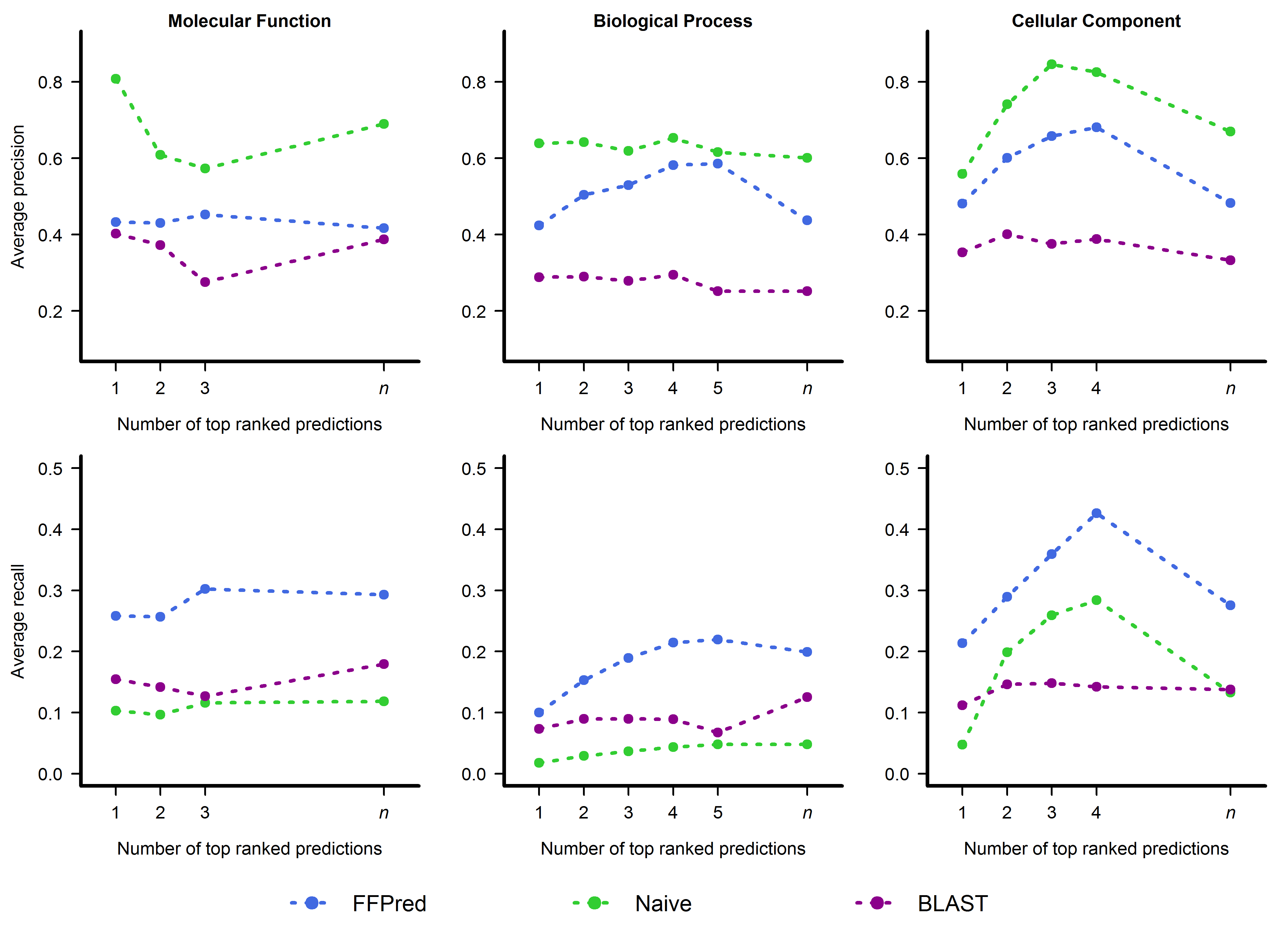


***Figure S1. Comparison of precision and recall values against number of top ranked predictions.***

The top row plots compare the average precision of the highest scoring GO term predictions by FFPred 3, Naïve and BLAST for the MF (left), BP (centre) and CC (right) sub-ontologies, respectively. The bottom row panels show the average recall for the same subsets of predictions as above. Data are plotted only when there are at least 25 targets with *x*∈{1,2,3,4,5} predictions and *x* validated annotations or more. The label *n* represents the case where for each protein the number of predictions assessed equals the number of experimentally supported functions.


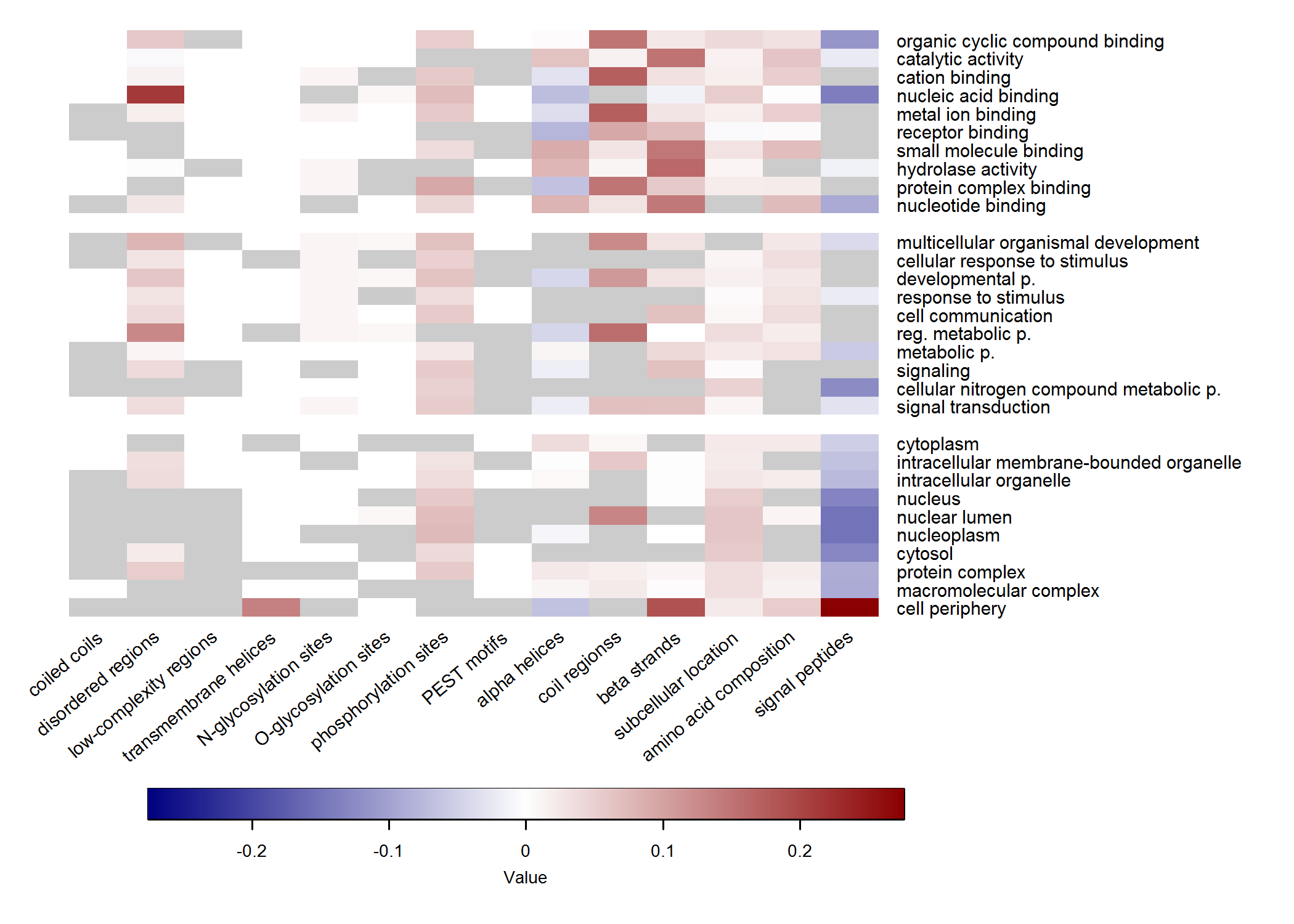


***Figure S2. Enrichment of biological features for the most conserved functions in human alternative isoforms.***

The heatmaps compare the feature values calculated for the annotated splice variants with those used to train the classifiers for each of the ten most conserved functions in the MF (top), BP (centre) and CC (bottom) domain, respectively – regardless of the expected accuracy. Warmer (colder) colours represent higher (lower) values in the human isoform proteome than in the positive training set for the corresponding GO term. Grey cells indicate feature groups not used by FFPred 3 to make predictions.


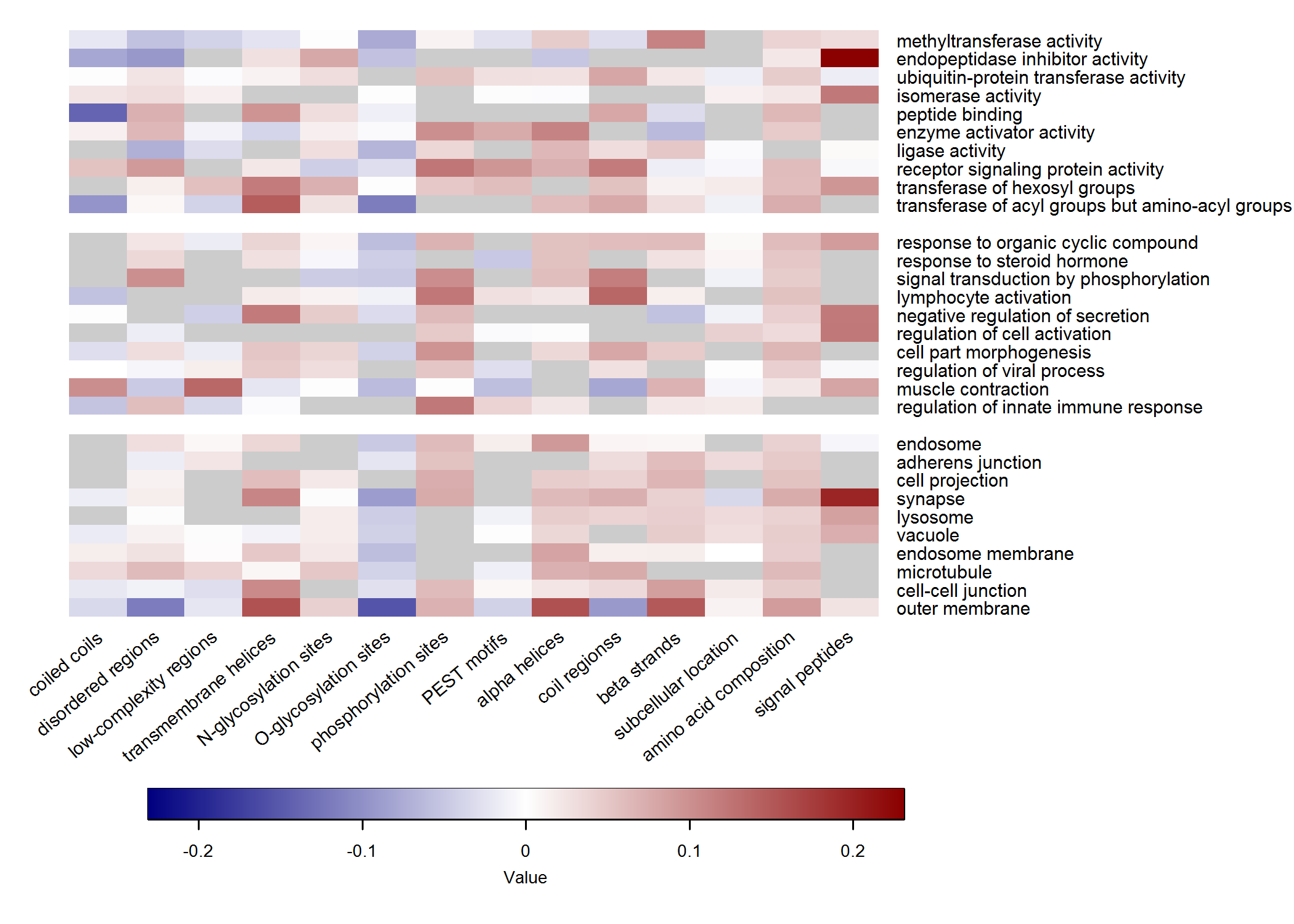


***Figure S3. Relationship between biological features and GO terms preferentially associated with main splice isoforms.***

For each function, the heatmaps report the correlation between the feature values calculated for the annotated splice variants with the estimated probability produced by the corresponding classifier. Only the ten GO terms with highest propensity for the canonical splice variants are listed for the MF (top), BP (centre) and CC (bottom) domain, respectively – regardless of the SVM expected accuracy. Warmer (colder) colours represent higher (lower) values of median correlation across each feature group. Grey cells indicate feature groups not used by FFPpred 3 to make predictions.


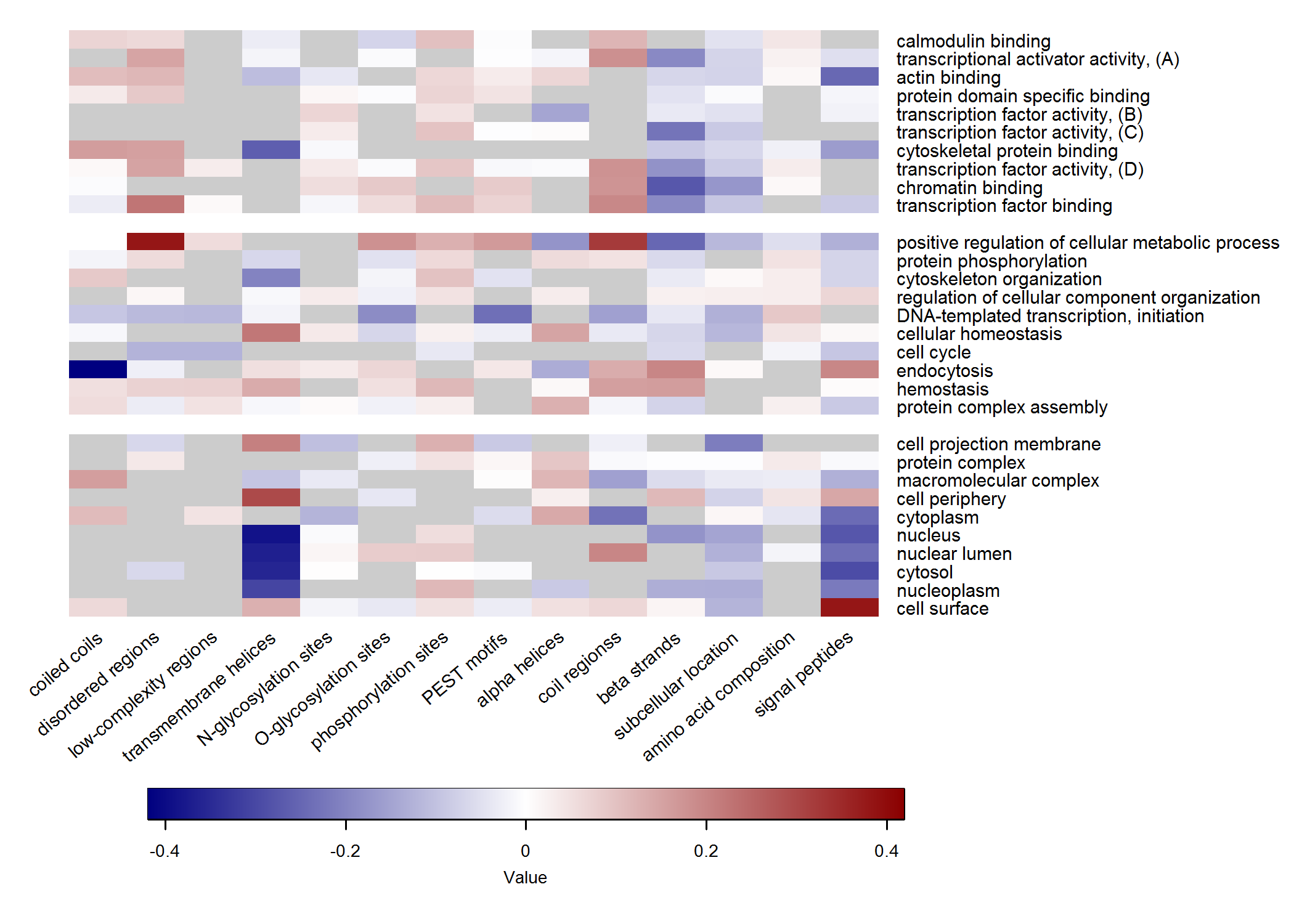


***Figure S4. Relationship between biological features and GO terms preferentially associated with alternative splice isoforms.***

For each function, the heatmaps report the correlation between the feature values calculated for the annotated splice variants with the estimated probability produced by the corresponding classifier. Only the ten GO terms with highest propensity for alternative variants are listed for the MF (top), BP (centre) and CC (bottom) domain, respectively – regardless of the predictor’s expected performance. Warmer (colder) colours represent higher (lower) values of median correlation in each feature group. Grey cells indicate feature groups not used by FFPpred 3 to make predictions.


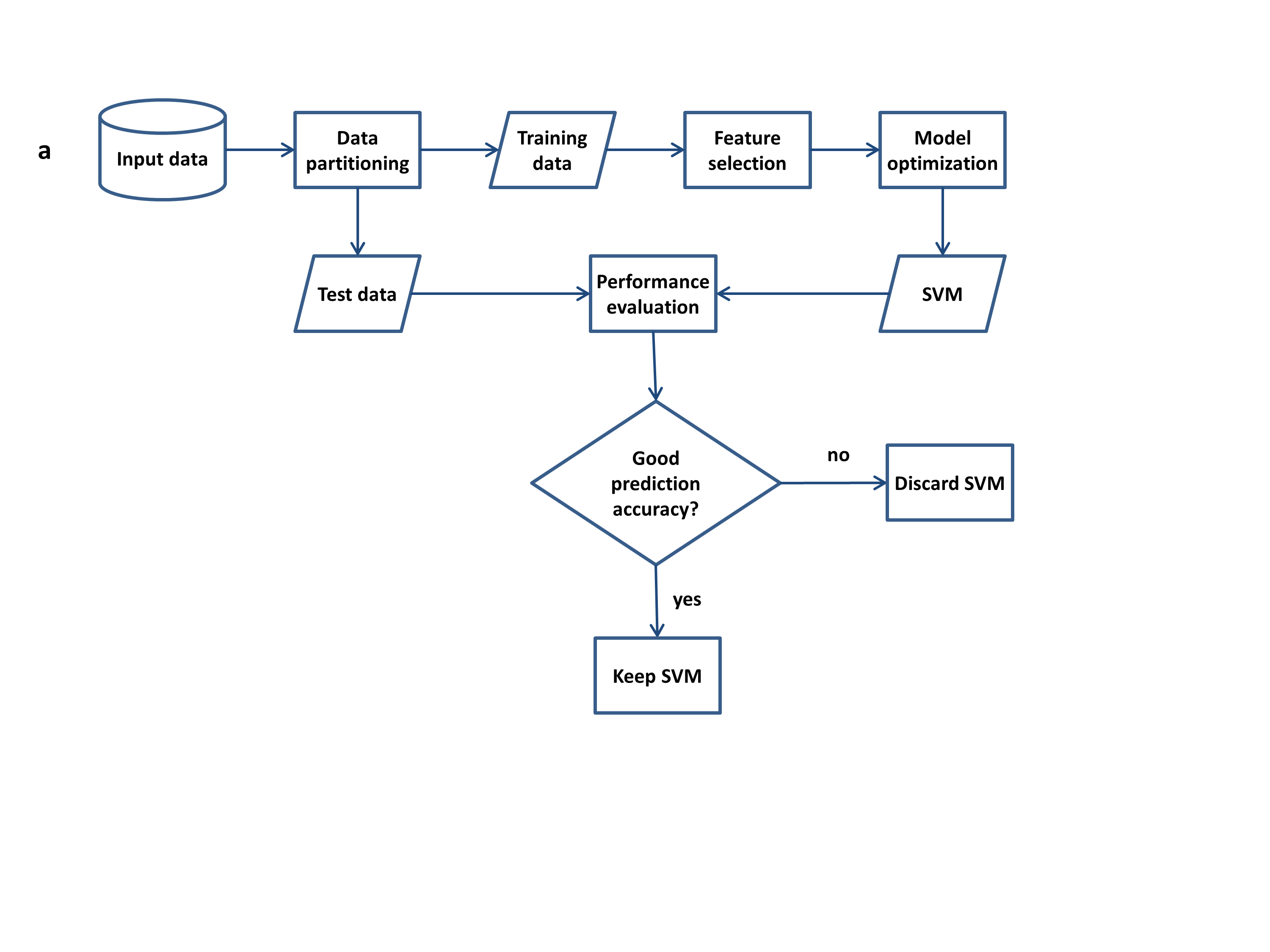


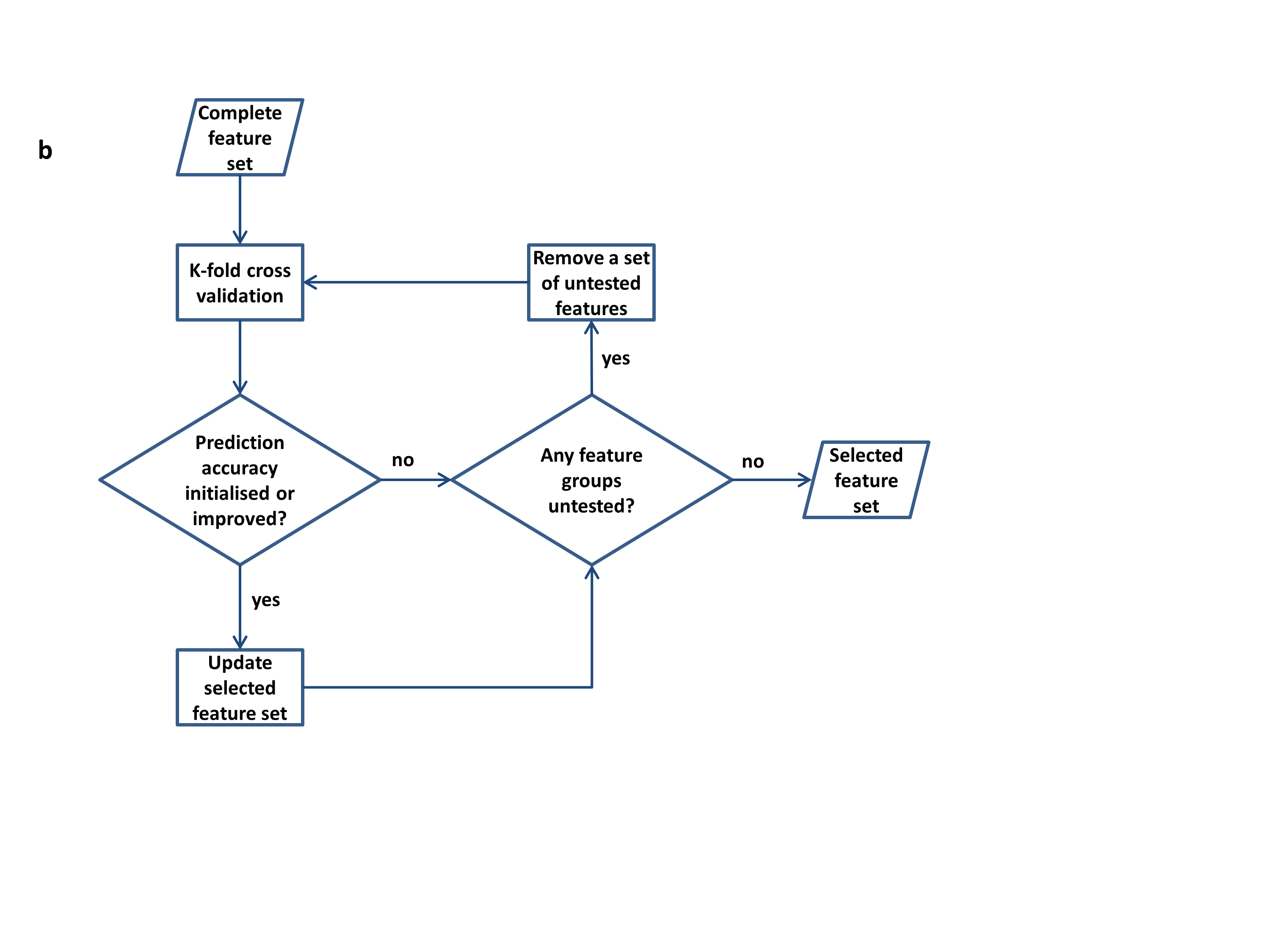


***Figure S5. Flowchart of FFPred 3 training process.***

For each GO term, a dedicated SVM is trained following the standard supervised learning procedures (a). Feature selection is performed via a recursive feature group elimination algorithm (b).

***Table S1. Comparison between FFPred* 3 top-ranked predictions and experimentally validated annotations.**

For each protein, the table includes the GO term assignments that have been curated by the UniProt-GOA team and compares them against the most confident predictions FFPred 3 made for each sub-ontology (CC: cellular component, MF: molecular function, and BP: biological process)

| **UniProtKB accession** | **GO annotations** | | | **FFPred 3 top-ranked predictions** | | |
| --- | --- | --- | --- | --- | --- | --- |
|  | **domain** | **Term** | **Name** | **term** | **name** | **score** |
| O60676 | BP | GO:0010951 | negative regulation of endopeptidase activity | GO:0010466 | negative regulation of peptidase activity | 0.702 |
| P19013 | CC | GO:0005634 | nucleus | GO:0005737 | cytoplasm | 0.783 |
|  | CC | GO:0009986 | cell surface | GO:0043229 | intracellular organelle | 0.795 |
|  | CC | GO:0045095 | keratin filament | GO:0005882 | intermediate filament | 0.804 |
|  | CC | GO:0045111 | intermediate filament cytoskeleton | GO:0045111 | intermediate filament cytoskeleton | 0.816 |
|  | BP | GO:0007010 | cytoskeleton organization | GO:0006996 | organelle organization | 0.647 |
| P86397 | CC | GO:0005730 | nucleolus | GO:0005737 | cytoplasm | 0.768 |
|  | CC | GO:0005737 | cytoplasm | GO:0043231 | intracellular membrane-bounded organelle | 0.733 |
|  | CC | GO:0005739 | mitochondrion | GO:0005739 | mitochondrion | 0.698 |
| Q5SSJ5 | CC | GO:0005634 | nucleus | GO:0043229 | intracellular organelle | 0.798 |
|  | CC | GO:0005694 | chromosome | GO:0005634 | nucleus | 0.790 |
|  | MF | GO:0003677 | DNA binding | GO:0003676 | nucleic acid binding | 0.813 |
|  | MF | GO:0031491 | nucleosome binding | GO:0003677 | DNA binding | 0.819 |
|  | BP | GO:0006355 | regulation of transcription, DNA-templated | GO:0006355 | regulation of transcription, DNA-templated | 0.680 |
|  | BP | GO:0042127 | regulation of cell proliferation | GO:0010468 | regulation of gene expression | 0.668 |
|  | BP | GO:0070828 | heterochromatin organization | GO:0044237 | cellular metabolic process | 0.656 |
|  | BP | GO:0071456 | cellular response to hypoxia | GO:0006351 | transcription, DNA-templated | 0.648 |
|  | BP | GO:0097298 | regulation of nucleus size | GO:0006996 | organelle organization | 0.641 |
|  |  |  |  |  |  |  |
|  |  |  |  |  |  |  |
| Q70CQ3 | CC | GO:0005741 | mitochondrial outer membrane | GO:0031966 | mitochondrial membrane | 0.800 |
|  | MF | GO:0004197 | cysteine-type endopeptidase activity | GO:0008233 | peptidase activity | 0.857 |
|  | MF | GO:0004843 | thiol-dependent ubiquitin-specific protease activity | GO:0008234 | cysteine-type peptidase activity | 0.842 |
|  | BP | GO:0000422 | mitophagy | GO:0006508 | proteolysis | 0.693 |
|  | BP | GO:0016579 | protein deubiquitination | GO:0044257 | cellular protein catabolic process | 0.691 |
|  | BP | GO:0035871 | protein K11-linked deubiquitination | GO:0051603 | proteolysis involved in cellular protein catabolic process | 0.684 |
|  | BP | GO:0044313 | protein K6-linked deubiquitination | GO:0030163 | protein catabolic process | 0.670 |
| Q96FV3 | MF | GO:0019899 | enzyme binding | GO:0042802 | identical protein binding | 0.209 |
|  | BP | GO:0072594 | establishment of protein localization to organelle | GO:0006810 | transport | 0.643 |
| Q96Q77 | MF | GO:0000287 | magnesium ion binding | GO:0005509 | calcium ion binding | 0.857 |
|  | MF | GO:0005509 | calcium ion binding | GO:0046872 | metal ion binding | 0.805 |
| Q9NX20 | CC | GO:0005743 | mitochondrial inner membrane | GO:0005759 | mitochondrial matrix | 0.827 |
|  | BP | GO:0006996 | organelle organization | GO:0006412 | translation | 0.707 |
|  | BP | GO:0070124 | mitochondrial translational initiation | GO:0010467 | gene expression | 0.700 |
|  | BP | GO:0070125 | mitochondrial translational elongation | GO:0044237 | cellular metabolic process | 0.697 |
|  | BP | GO:0070126 | mitochondrial translational termination | GO:0044267 | cellular protein metabolic process | 0.674 |
| Q9ULR3 | CC | GO:0005634 | nucleus | GO:0043231 | intracellular membrane-bounded organelle | 0.787 |
|  | CC | GO:0005737 | cytoplasm | GO:0005737 | cytoplasm | 0.784 |
|  | MF | GO:0004721 | phosphoprotein phosphatase activity | GO:0004721 | phosphoprotein phosphatase activity | 0.874 |
| Q9Y221 | CC | GO:0005634 | nucleus | GO:0005737 | cytoplasm | 0.797 |
|  | CC | GO:0005730 | nucleolus | GO:0005829 | cytosol | 0.733 |
|  | CC | GO:0005737 | cytoplasm | GO:0043231 | intracellular membrane-bounded organelle | 0.644 |
|  | MF | GO:0044822 | poly(A) RNA binding | GO:0003676 | nucleic acid binding | 0.813 |
